# Snacking on Television: A Content Analysis of Adolescents’ Favorite Shows

**DOI:** 10.5888/pcd13.160014

**Published:** 2016-05-19

**Authors:** Marla E. Eisenberg, Nicole I. Larson, Sarah E. Gollust, Dianne Neumark-Sztainer

**Affiliations:** Author Affiliations: Nicole I. Larson, Dianne Neumark-Sztainer, Division of Epidemiology and Community Health, University of Minnesota School of Public Health, Minneapolis, Minnesota; Sarah E. Gollust, Division of Health Policy and Management, University of Minnesota School of Public Health, Minneapolis, Minnesota.

## Abstract

**Introduction:**

Snacking is a complex behavior that may be influenced by entertainment media. Research suggests that snacking and unhealthy foods are commonly shown in programming that targets young audiences, but shows selected for study have been limited. We conducted a content analysis on shows that were named as favorites by adolescents to characterize portrayals of snacking on popular television.

**Methods:**

A diverse sample of 2,130 adolescents (mean age, 14.3 y) listed 3 favorite television shows in a 2010 school-based survey. Three episodes each of the 25 most popular shows were coded for food-related content, including healthfulness, portion size, screen time use, setting, and social context. We also analyzed the characteristics of characters involved in eating incidents, the show type, and the show rating. We used χ^2^ tests, binomial tests, and multilevel regression models to compare incidence of snacks versus meals, the characteristics of those involved, and snacking across show characteristics.

**Results:**

Almost half of food incidents on television shows were snacks. Snacks were significantly more likely than meals to be “mostly unhealthy” (69.3% vs 22.6%, *P* < .001) and were more likely to include screen time use (25.0% of snacking incidents vs 4.0% of meals, *P* < .001). Young characters and those coded as being of low socioeconomic status or overweight were overrepresented in snacking incidents. Sitcoms and shows rated for a youth audience were significantly more likely to portray snacking than were shows for adult audiences.

**Conclusion:**

Media awareness and literacy programs should include foods and snacking behaviors among the issues they address. More healthful portrayals of food and dietary intake in entertainment shows’ content would create a healthier media environment for youth.

## Introduction

Snacking is a complex behavior that has many definitions in research, including eating between main meals and eating typical snack foods, such as chips, candy, and fruit-flavored drinks (1). National survey data indicate that most adolescents consume 2 or more snacks between meals per day, and energy-dense, nutrient-poor food and beverages are major sources of the energy consumed as snacks (2,3). Research has established that dietary intake is influenced by multiple physical and social cues in young people’s home and school environments (4) and by advertising (5). However, little research has examined influences on snacking behaviors in particular, especially in the domain of media (6).

According to a 2015 report, adolescents spend an average of 17 hours per week watching television (7), and media messages are a powerful social influence (8). Research on health behaviors such as smoking, early sexual activity, and violence has identified links between viewing entertainment media content and enacting these behaviors (8–11). Children and adolescents may form “pseudo-friendships” with television characters and look to them as behavioral role models (12). Mass media also powerfully affects perceptions of peer norms, which are strong influences on adolescent behaviors (11,13). The strength of influence may further depend on the salience of the role model, such as whether the television character matches the viewer in terms of sex or race or has desired attributes (eg, slim body type).

Existing research on the portrayal of food on television has focused almost exclusively on advertising and has suggested associations between food marketing and children’s dietary intake and weight (5). However, programming itself accounts for a far greater proportion of viewing time than do advertisements. New technology increasingly permits viewing without advertising (which has led to an increase in product placement within shows), giving programming content greater relevance. Little information is available on the portrayal of foods on popular television shows, and most studies are outdated (14–19). However, a small body of recent literature found that snacking and unhealthy snack foods are commonly shown in programming that targets young audiences (19–21).

A limitation of recent studies is the sample of media content selected for analysis. Researchers selected programming on the basis of its intended audience (ie, children), which may differ from what young people actually watch (22). Therefore, viewers’ actual exposure to unhealthy content may be inaccurately estimated by studies restricted to youth programming. Similarly, existing studies restricted their parameters to single television networks (eg, Disney channel [19–21]) that do not represent the breadth of content available to youth.

This study builds on the limited extant literature on the relationship between media programming and snacking behaviors among youth, using an innovative design. Write-in survey data were collected from a diverse population-based sample of adolescents to identify favorite programs, and popular programs were content-analyzed. The main research objective was to characterize portrayals of snacking, including frequency, healthfulness, portion size, and characters’ use of screens while consuming food. Snacking was compared with eating meals as a reference point for the portrayal of food on television. Because on-screen behaviors may differ for population subgroups and because some characters may be relatable or desirable for young viewers, we also examined the demographic characteristics and weight status of the characters involved. Finally, we examined snacking portrayals by show type (eg, sitcom) and by intended audience. Understanding ways in which snacking behavior is portrayed in popular television will inform recommendations for families and future study of associations with eating patterns among youth.

## Methods

### Population, setting, and design

In 2010, 2,793 adolescents attending public middle schools and high schools in Minneapolis/St. Paul, Minnesota, completed surveys as part of a cross-sectional, population-based study of weight and related behaviors (EAT 2010) (23). Participants were split among middle school (grades 6–8; 46.1%) and high school (grades 9–12; 53.9%). Almost half were male (46.8%), and the sample was racially and ethnically diverse (18.9% white, 29.0% African American, 19.9% Asian American, 16.9% Hispanic, 3.7% Native American, and 11.6% mixed or other race), reflecting the demographic profile of participating schools (24).

Participants were asked to write in the titles of their 3 favorite television shows. One or more favorite shows were named by 2,130 participants (mean age, 14.3 y [standard deviation, 2.0 y]), yielding 653 unique shows. Favorite shows were ranked by weighting each participant’s first listed show more highly than the second show, which counted more than the third show. We excluded entries that were broad topic areas (eg, sports, music videos), networks (eg, MTV), or sports or music events (eg, *106 & Park*) and included only shows with characters, scenes, dialogue, and plot. Closely related shows such as *CSI*, *CSI: NY*, and *CSI: Miami* were combined and considered as the original version (22). The 25 most popular shows were content analyzed (Appendix); 54.8% of the EAT 2010 sample listed 1 or more of these top 25 shows.

Three episodes of each show were randomly selected from the 2010 season and were accessed via online services (eg, network website, Netflix). Coding was done in 2 batches of 3 coders for the first 10 shows and 2 coders for the remaining 15 shows, with 1 original coder training the 2 new coders. Interrater reliability was ascertained for each batch. The University of Minnesota’s Institutional Review Board’s Human Subjects Committee approved all protocols used in EAT 2010 and determined that this analysis was exempt from review.

### Coding instrument and variables

A coding instrument was created on the basis of previous research for EAT 2010 to assess on-screen food-related incidents, characters traits, and show characteristics (14,16,17). The team developed and revised the coding instrument in multiple iterations and pilot tested it with episodes of the same shows from other seasons before it was finalized. An accompanying codebook was used to detail assessment of each item to enhance consistency across coders.

Coders recorded any time a food was shown or referenced on screen (ie, a *food incident*), and 6 measures were the focus of this analysis. We employed a definition of snacking as a food incident that was not part of a meal. *Incident type* indicated whether a food incident was breakfast, lunch, dinner, or a snack. Several on-screen cues were used to identify meals, including time of day (eg, food eaten in a cafeteria during the school day was coded as lunch), number of foods (eg, multiple food items served at a table), dialogue spoken (eg, “Another dinner got away from you?”), and other context (eg, table set for a family). Food incidents outside of meals were coded as snacks. When typical snack foods were eaten during a meal (eg, chips with a burger), the incident was coded as a meal. Of the 559 food incidents coded across 75 episodes, type could not be determined (eg, foods shown in the background of a scene) for 163 (29.2%), and these were excluded from analysis. 

The overall *healthfulness* of a food incident was coded as mostly healthy (eg, well balanced meals, fruit, vegetables, lean proteins, cheese, yogurt), mostly unhealthy (eg, baked desserts, candy, potato chips, snack foods, sugared cereal), or unclear (typically when an incident was referenced rather than shown). When multiple foods were shown, the incident was coded for the overall balance (eg, “chicken, corn, mashed potatoes, milk, crumble, undeterminable item” was coded as mostly healthy; “pretzel snack bags, cake, ketchup, mustard, other undeterminable food” was coded as mostly unhealthy). If foods were shown being eaten by a character, coders noted whether the portion was *excessive* (ie, much more than is appropriate for the given meal type, for example, with food heaping over the plate or a character taking multiple servings). Examples include “bucket of cheese,” “plate full of bacon smothered in pancake syrup, sausage links, milk.”

Non–food-related aspects of each incident were also coded. *Screen time* was coded as a television, computer, or similar electronic device in use during the incident. Two additional items were used to describe food incidents, including the physical *setting* (eg, eating at school, eating at a table at home) and the *social context* (eg, eating alone or with others).

Two show characteristics were used in this analysis. *Show type* was coded as sitcom, cartoon, or drama. Parental Guide *ratings* were ascertained on the basis of information taken from the show’s website or the Internet Movie Database website (www.imdb.com) and were combined into 3 categories for analysis: youth audience (TV-Y and TV-G), general audience (TV-PG), and older audience (TV-14 and TV-MA). 

Characteristics of the main and supporting characters in each show were recorded (22). Briefly, characters’ socioeconomic status (SES) was coded as “poor/lower class,” indicated by material goods (eg, cars, clothing, housing) or references to Medicaid, food shortage, or other needs of low-SES people; or “wealthy/upper class,” indicated by material goods (eg, a known celebrity, expensive car, large home) or references to extreme wealth. All other characters were coded as “average/middle class.” A character’s weight status was coded as “thin/underweight” if the character appeared thinner than normal with obvious clavicle bones, facial bones, rib cage, or other bones protruding; if the character’s body mass index (BMI) were calculated, it would be less than 18.5 kg/m^2^. A character’s weight status was coded as “overweight” if the character had excess body fat (eg, obvious pot belly); if the character’s BMI were calculated, it would likely be from 25.0 to 30.0 kg/m^2^. A character’s weight status was coded as “obese” if the character carried an excessive amount of weight; if the character’s BMI were calculated, it would likely be more than 30 kg/m^2^. All other characters were coded as “average weight.”

### Data collection

The data collection protocol was identical in both batches of coding (shows 1–10 and 11–25). Each episode was coded using a 3-step viewing process. First, coders identified all incidents to be coded and noted characters involved in each relevant scene. Second, coders entered all relevant information into the coding instrument. A third and final viewing ensured that all information was captured and appropriately coded.

Intercoder reliability was calculated at the end of the coder training periods, using episodes of select shows in a previous season, and was completed for the 2 batches separately. Cohen’s κ statistic is appropriate for categorical variables and adjusts for chance agreement (25). In the first batch, individual items that had a κ of less than 0.70 were reviewed by the team and revised to finalize the coding instrument. Discrepancies during the training of the second set of coders were resolved by the lead coder from the first group. Food and beverage items from shows 1 through 10 had a mean κ of 0.73, and food and beverage items from shows 11 through 25 had a mean κ of 0.98. Character demographics from shows 1 through 10 had a mean κ of 0.81, and character demographics from shows 11 through 25 had a mean κ of 1.00, indicating excellent reliability (25).

### Data analysis

Four data sets were created to address this study’s research questions: favorite television shows (n = 25), food incidents (n = 396), main and supporting characters (n = 366), and a combined data set of characters involved in food incidents (n = 971). For example, if 3 characters were shown eating a meal together, this single incident would generate 3 separate cases, 1 for each character.

We used these data sets separately and in combination for analysis; we used χ^2^ tests to compare snacks with meals in favorite television shows and tested differences in the characters involved in snacking versus meal incidents. In addition, 2-sided binomial tests were used to test differences in the characteristics of those involved in snacking incidents compared with the overall sample of characters across all shows. Finally, multilevel regression models were used to test associations between snacking characteristics and show characteristics, clustering food incidents within the same show to permit accurate inference. Predicted probabilities of each snacking characteristic were generated from these models. Analyses were conducted using SAS version 9.3 (SAS Institute, Inc). Significance was set at an ɑ level of .05.

## Results

### Snack versus meal portrayals

Food incidents were common in this sample of shows; 92.0% of coded episodes included at least 1 incident (mean = 5.3 incidents/episode; range, 0–17). Almost half of food incidents (48.5%) were snacks and the rest were breakfasts (11.1%), lunches (14.4%) or dinners (26.0%). Food incidents coded as snacks were significantly more likely to be “mostly unhealthy” than foods coded as meals (69.3% vs 22.6%, *P* < .001) (Table 1).

**Table 1:** Characteristics of Food Incidents (N = 252) Portrayed in Adolescents’ 25 Favorite Television Shows, Minnesota, 2010

Characteristic	Snacks, %	Meals, %	χ^2^	*P* Value
**Healthfulness^a^**				
Mostly healthy	23.4	52.9	88.2	<.001
Undeterminable	7.3	24.5		
Mostly unhealthy	69.3	22.6		
**Excessive portion size^b^**				
No	90.0	92.0	0.16	.69
Yes	10.0	8.0		
**Included screen time use^b^**				
No	75.0	96.0	12.7	<.001
Yes	25.0	4.0		
**Physical setting^b^**				
At school	3.3	6.7	45.1	<.001
At a table (at home)	3.3	40.0		
At home (but not at table)	33.3	10.7		
At a sit-down restaurant	13.3	26.7		
At a fast-food restaurant	3.3	1.3		
At work	11.7	9.3		
“On the run”	0.0	0.0		
Other	31.7	5.3		
**Social context^b^**				
Alone	16.7	13.3	8.0	.05
With peers	71.7	54.7		
With family member(s)	11.7	30.7		
Other	0	1.3		

a Overall healthfulness of a food incident was coded as mostly healthy (eg, well balanced meals, fruit, vegetables, lean proteins, cheese, yogurt), mostly unhealthy (eg, baked desserts, candy, potato chips, snack foods, sugared cereal), or unclear (typically when an incident was referenced rather than shown). When multiple foods were shown, the incident was coded for overall balance.

b Of 135 incidents of food consumed on screen.

Among the 396 food incidents, 63.6% (n = 252) were shown, and 36.4% (n = 144) were referenced or mentioned by characters but not shown. Of the incidents of food shown on screen, 135 involved food shown being consumed by a character, and significant differences were noted between snacks and meals (Table 1). For example, 25% of snacking incidents included screen time (ie, television or computer use while eating), compared with only 4.0% of meal incidents (*P* < .001). There were no differences in the proportion of incidents involving excessive portion sizes in snacks versus meals.

### Characters involved in food incidents

Table 2 shows the descriptive demographic and weight status variables of all main and supporting characters in the top 25 favorite shows (n = 366). More than half of characters were male (58.9%) and most were adults. Most characters were coded as white (76.8%) and as being of average SES (81.1%). Most were coded as thin or of average weight (86.0%). 

**Table 2:** Characteristics of Characters (N = 366) Involved in Food Incidents (N = 971) in Adolescents’ 25 Favorite Television Shows, Minnesota, 2010

Characteristic	Total %	Snack Incidents, %	Meal Incidents, %
**Sex**			
Male	58.9	62.8	60.6
Female	41.1	37.2	39.4
Snacks vs meals: χ^2^, *P* value	0.42, .52		
Snacks vs total: *z* score, *P* value	1.59, .11		
**Age group**			
Child/adolescent (<20 y)	32.5	41.8	32.2
Young adult (20–29 y)	17.2	13.4	17.9
Adult (≥30 y)	50.3	44.8	49.9
Snacks vs meals: χ^2^, *P* value	9.14, .01		
Snacks vs total: *z* score, *P* value	3.98, <.001		
**Race/ethnicity**			
White	76.8	80.2	78.9
Other	23.2	19.8	21.1
Snacks vs meals: χ^2^, *P* value	0.22, .64		
Snacks vs total: *z* score, *P* value	1.60, .11		
**Socioeconomic status^a^**			
Poor/lower class	4.0	9.4	3.5
Average/middle class	81.1	72.7	71.2
Wealthy/upper class	14.9	17.9	25.3
Snacks vs meals: χ^2^, *P* value	17.3, <.001		
Snacks vs total: *z* score, *P* value	5.56, <.001		
**Weight^b^**			
Thin/underweight and average weight	86.0	87.0	90.9
Overweight	9.6	10.3	5.6
Obese	4.4	2.7	3.5
Snacks vs meals: χ^2^, *P* value	6.78, .03		
Snacks vs total: *z* score, *P* value	0.59, .28		

Abbreviation: BMI, body mass index.

a Characters’ socioeconomic status (SES) coded as “poor/lower class,” indicated by material goods (eg, cars, clothing, housing) or references to Medicaid, food shortage, or other needs of low-SES people. Characters coded as “wealthy/upper class,” indicated by material goods (eg, a known celebrity, expensive car, large home) or references to extreme wealth. All other characters coded as “average/middle class.”

b A character’s weight status was coded as “thin/underweight” if the character appeared thinner than normal with obvious clavicle bones, facial bones, rib cage, or other bones protruding; if the character’s BMI were calculated, it would be <18.5 kg/m^2^. A character’s weight status was coded as “overweight” if the character had excess body fat (eg, obvious pot belly); if the character’s BMI were calculated, be 25.0–30.0 kg/m^2^. A character’s weight status was coded as “obese” if the character carried an excessive amount of weight; if the character’s BMI were calculated, it would likely be >30.0 kg/m^2^. All other characters were coded as “average weight.”

Two comparisons were made using the 971 distinct character-food incidents: 1) the demographic and weight characteristics of those involved in snack versus meal incidents, and 2) the demographic and weight characteristics of those involved in snack incidents compared with the total sample of 366 characters (Table 2). Several significant differences emerged: 32.2% of characters in meal incidents were children or adolescents, but 41.8% of those in snack incidents were in these age groups (*P* = .01). The proportion of snack incidents that included children or adolescents (41.8%) was also significantly higher than the overall proportion of child and adolescent characters (32.5%, *P* < .001). Involvement in snacks versus meals also differed by characters’ apparent SES; 9.4% of snacking incidents including characters coded as lower SES, compared with 3.5% of meal incidents (*P* < .001) and 4.0% of characters overall (*P* < .001). Overweight characters were shown in snacking incidents at almost twice the rate of meal incidents (10.3% vs 5.6%, *P* = .03) but were not overrepresented in snacking scenes compared with the total sample of characters (9.6%).

### Differences across shows

Sitcoms were significantly more likely than other types of shows to portray snacking, and shows rated for a general or a youth audience were more likely to show snacking than shows intended for adult audiences (Table 3). Cartoons were significantly more likely to portray excessive consumption of snacks (37.5%) than sitcoms (2.8%) or dramas (0%; *P* = .05). There were no significant differences in excessive consumption of snacks or screen time with snacks across show types or ratings.

**Table 3:** Show Characteristics and Predicted Probabilities of Each Snack Characteristic in Adolescents’ 25 Favorite Television Shows, Minnesota, 2010^a^

Characteristic	Snacks vs Meals	Mostly Unhealthy Snacks	Excessive Consumption of Snacks^b^	Screen Time Use With Snacks^b^
	%			
**Type**				
Sitcom (n = 12)	57.5^c^	65.1	2.8^c^	28.5
Cartoon (n = 4)	29.9^d^	87.2	37.5^d^	27.3
Drama (n = 9)	40.2^d^	71.3	0.0^c^	9.4
F statistic, *P* value	4.81, .009	1.05, .35	3.28, .05	0.67, .52
**Rating^e^**				
Youth (Y or G) (n = 5)	52.4^c^^,^ d	56.3	22.8	15.6
General (PG) (n = 8)	57.3^c^	75.5	4.1	31.6
Mature (14 or MA) (n = 12)	36.7^d^	71.9	0	19.6
F statistic, *P* value	3.29, .04	1.48, .23	1.42, .26	0.57, .57

a From multilevel regression models, accounting for clustering of incidents within shows.

b Of 135 incidents of food consumed on screen.

c,d Predicted probabilities that share a superscript are not significantly different (*P* > .05).

e Ratings were ascertained on the basis of information taken from each show’s website or the Internet Movie Database website (www.imbd.com) and combined into 3 categories for analysis: “Y or G,” youth audience; “PG,” general audience; and “14 or MA,” older audience.

## Discussion

In this sample of popular shows, snacks were shown often and typically included unhealthy foods or behaviors, such as watching television while eating. Snacking incidents disproportionately included characters in certain demographic groups (eg, youth, low-SES) and overweight characters. Snacking was also more common in sitcoms than in other types of shows and less common in shows that target adult audiences. Our findings generally align with the limited number of recent studies showing a high prevalence of snacking, unhealthy foods, and poor dietary patterns in entertainment programming (19–21), but they are the first in a sample of shows named as popular by youth. Young people experience a media environment that normalizes unhealthy foods and snacking behaviors, which is expected to contribute to unhealthy eating behaviors among viewers.

Although snacking behaviors on television shows may seem innocuous, extensive research has demonstrated that on-screen behaviors create social norms of behavior that are then seen as typical or expected by viewers (5,10). Characters may be seen as pseudo-friends and role models (10,26). Peer modeling raises concerns that frequent viewing of characters snacking, particularly on unhealthy food items, may contribute to these same behaviors in viewers when they are seen as normative for those with similar demographic characteristics or desirable body types. Furthermore, frequent unhealthy snacking by thin or average-weight characters may set up unrealistic expectations about dietary behaviors and weight gain over time. More frequent snacking among children and adolescents on television (and higher rates of snacking in shows that target youth audiences) may create a norm of snacking, especially among young characters who may be the most salient on-screen “peers” to young viewers. Similarly, characters coded as lower income were more often involved in snacking incidents. Such images contribute to expectations of poor dietary intake in populations that already have a high prevalence of obesity (26) and may promote negative stereotypes (27).

This study has several strengths. The shows analyzed were listed as favorites by a diverse sample of adolescents. This sampling method allowed us to analyze television shows that were actually popular among adolescents rather than those that simply target adolescent audiences. In addition, coding 3 episodes of 25 shows identified a large number of food incidents, which permitted statistically valid comparisons across incident, character, and show characteristics.

This study also has limitations. First, certain types of entertainment programming could not be meaningfully coded, such as sports shows or music video shows. Scripted shows (the focus of this study) make up a large portion of the television schedule, but other formats likely include portrayals of food and food-related behaviors (18). Similarly, less popular shows — nominated as favorites by almost half the student sample — were not included in this analysis. Findings may therefore not apply to all shows that youth favor. Second, snacking is difficult to define; the type of food, the quantity of food consumed, the time of day it was consumed, and other factors contribute to the interpretation of whether the food eaten was a snack or a meal (28,29). Although interrater reliability was high for this item (κ = 0.98 in both batches) and television content creators rely on easily interpretable cues to establish a scene (eg, a set table to indicate a meal), viewers may understand eating instances differently, on the basis of their experiences. Finally, favorite shows were nominated in 2010 and portrayals of snacking may have changed in recent years.

Findings provide the basis for continued research that examines on-screen unhealthy snacking behaviors in relation to eating among youth. Detailed exploration is needed of ways in which youth identify with particular characters, perceive on-screen behaviors as normative, and adapt their behavior to understand underlying mechanisms. Both the content of entertainment media and the advertising between segments should be routinely included in studies of media influence on health behaviors.

Findings on the portrayal of unhealthy snacking add to research on media content (8–11), which has led to recommendations for reduced screen time, parental coviewing with youth, and media regulation. Media awareness and literacy programs should include foods and snacking behaviors among the issues they cover, to break the links between on-screen behavior, perceived norms, and health behaviors. Within the entertainment industry, adoption of voluntary guidelines for more healthful portrayals of dietary intake in show content, as has been done for smoking depictions, would parallel industry changes in advertising and create a healthier media environment for youth.

## Appendix Top 25 Shows Nominated by EAT 2010 Participants

1. *Family Guy*

2. *The Simpsons*

3. *SpongeBob Square Pants*

4. *CSI*

5. *iCarly*

6.* South Park*

7. *Two and a Half Men*

8. *That ’70s Show*

9. *The Game*

10. *George Lopez*

11. *Everybody Hates Chris*

12. *House*

13. *The Vampire Diaries*

14. *My Name is Earl*

15. *Gossip* *Girl*

16. *The Office*

17. *Degrassi*

18. *Hannah Montana*

19. *Wizards of Waverly Place*

20. *The Suite Life on Deck*

21. *Secret Life of the American Teenager*

22. *Supernatural*

23. *NCIS*

24. *Bones*

25. *Scrubs*
